# Joint interprofessional education of pharmacy and dietetics undergraduates - a scoping review

**DOI:** 10.1186/s12909-024-05411-4

**Published:** 2024-05-22

**Authors:** Anna Rudzińska, Piotr Guzy, Agnieszka Skowron, Jerzy Gąsowski, Karolina Piotrowicz

**Affiliations:** 1 Department of Internal Medicine and Gerontology, Faculty of Medicine, Jagiellonian University Medical College, 2 Jakubowskiego St., building I, 5th floor, 30- 688, Kraków, Poland; 2 Faculty of Pharmacy, Jagiellonian University Medical College, 30-688 Krakow, Poland

**Keywords:** Medical education, Interprofessional, Dietetics, Nutrition, Pharmacy, Undergraduates

## Abstract

**Introduction:**

Interprofessional education (IPE) is an integrative approach that enables collaboration of students of two or more different health professions in aim to acquire skills and competencies related not only to their field of study but also to ensure the standard of care based on collaborative practice. IPE has not yet been explored in relation to collaboration between dietetics-nutrition and pharmacy students, while there is evidence that in many cases nutrition is complementary to pharmacotherapy in the treatment process.

**Aim:**

The aim of this scoping review was to gather, describe and discuss all relevant literature regarding joint interprofessional training of pharmacy and dietetics-nutrition undergraduates.

**Methods:**

We performed a literature search for studies where IPE between dietetics-nutrition and pharmacy students was described. 2204 articles on this topic were identified. After eligibility assessment, 8 articles were included in the review.

**Results:**

Eight studies were included in the review. Two of these described IPE activities between dietetics and pharmacy students only. The included studies varied in setting, methodology and outcome measures and covered a wide range of topics relevant to clinical practice, such as management of inflammatory bowel diseases, care of the older adults or counselling skills. The most common teaching method was the use of case studies. Some of the included studies did not identify specific learning objectives. The most common way of gathering feedback from participants was through questionnaires and interviews.

**Conclusions:**

IPE of pharmacy and dietetics-nutrition students is feasible and may be beneficial in many aspects related to learning. However, there is no well-established model or standard that would facilitate the implementation of such activities in individual educational institutions.

## Background

Collaborative, interprofessional healthcare should become the model for healthcare delivery. According to the World Health Organization (WHO) document published in 2010, the benefits of interprofessional education (IPE) and interprofessional collaborative practice include shorter hospital stays, lower rates of complications and reduced mortality [1]. IPE is a topic of interest for research on graduate-level education in various aspects of medical care. However, literature reports vary in the models of educational approach evaluated, including, but not limited to, the number of different professions or specialties included, the educational level of participants (graduate or undergraduate), the learning settings, and the educational topics [2–5]. There are also significant differences in the effects measured across studies. These may aim to measure students’ knowledge, skills, or opinions and experiences, as well as clinical skills or effects on the functioning of care systems [2, 4, 5]. Student-oriented outcomes include effects related to specific clinical or professional areas, as well as general collaborative skills, including teamwork or communication skills [4, 6, 7].

In recent years, there has been a growing emphasis on incorporating elements of interdisciplinary education into the curricula of medical schools. This has been highlighted by the accreditation committees of medical and nursing schools in the United States, such as the Liaison Committee on Medical Education (LCME) [8] and the Accreditation Commission for Education in Nursing (ACEN) [9], which include in their guidelines requirements for teaching aimed at effective collaboration between different professions. The Accreditation Council for Pharmacy Education (ACPE) publishes guidelines for accreditation that include a requirement to introduce interprofessional activities aimed at teaching skills such as conflict resolution and recognition of different professional roles [10]. In the UK, the General Medical Council requires medical schools to provide opportunities for students to work with other health and social care professionals during the course of their studies [11].

There are well-documented examples of good practice in providing such learning activities for the aforementioned majors, while care teams in both hospital and community settings are becoming increasingly multi-professional [12]. Nowadays, pharmacological and nutritional lifestyle interventions are considered important and complementary treatment modalities and pharmacists and dietitians are becoming more common members of these care teams. This creates an area for collaborative learning between dietitian-nutritionists and pharmacists, which may be considered beneficial in training on topics relevant to clinical practice where the required competencies are cross-disciplinary and part of the curriculum overlaps. This approach of combining pharmacological and dietary interventions is reflected in the clinical guidelines for diabetes [13–15], hypertension [16], dyslipidaemia [17, 18], chronic kidney disease [19, 20] or exocrine pancreatic insufficiency [21, 22].

The two curricula have in common not only the learning outcomes related to knowledge of therapeutic interventions, but also the role of both professions in the health care system. Both dieticians and pharmacists are responsible for delivering elements of health education in many European countries. Tasks that used to be carried out mainly by doctors and nurses are now largely carried out by members of both professions. This creates favourable conditions for learning using interprofessional education methods. Dietetics and pharmacy students can transfer knowledge on chronic disease management to each other and support each other in acquiring skills for effective communication with other members of the healthcare team and, most importantly, with the patient. Such an approach at the undergraduate level can lay a solid basis for future professional collaboration.

The purpose of this scoping review is to gather, describe and discuss all relevant literature regarding joint interprofessional training of pharmacy and dietetics-nutrition undergraduates with particular focus on learning settings, methods, topics, and outcome measures of joint learning used in research.

## Methods

We used the extended definition of IPE proposed by Centre for the Advancement of Interprofessional Education (CAIPE), according to which IPE can be defined as occasions when members or students of two or more professions learn with, from and about each other to improve collaboration and the quality of care and services [23].

We decided to conduct this review in accordance with scoping review methodology, following PRISMA Extension for Scoping Reviews [24].

### Inclusion/exclusion criteria

We included each study that examined the interprofessional education initiatives involving pharmacy students and dietetics-nutrition students.

We excluded studies where:

- students of either pharmacy or dietetics-nutrition were not included;
- the majority (> 50%) of the group were postgraduates;
- it was uncertain, whether dietetics-nutrition and pharmacy students had the opportunity to work together;
- described learning outcomes of interprofessional learning activity were unrelated with future working environment and patient care (e.g. language courses, time management training).

During the screening stage, we considered only publications in English and Polish. We excluded narrative reviews, conference abstracts, letters, opinions, and editorials.

### Search strategy

We conducted systematic search of 3 medical databases: Medline (via PubMed), Cochrane Library and Embase with following queries:

- For PubMed and Cochrane: (((((((((((dietician) OR (nutritionist)) OR (dietitian)) OR (dieticians)) OR (nutritionists)) OR (dietitians)) OR (dietetics student)) OR (dietetics students)) OR (“Dietetics“[Mesh]))) AND ((((((pharmacist) OR (pharmacists)) OR (Pharmacy student)) OR (Pharmacy students)) OR (“Students, Pharmacy“[Mesh]))))
- For Embase: ((‘pharmacist’/exp OR pharmacist) OR ‘pharmacy education’/exp OR ‘pharmacy student’/exp) AND ((‘dietitian’/exp OR ‘dietitian’) OR ‘dietetics’/exp OR ‘dietetics student’/exp).

Search results are current as of May 17, 2023.

The selection of relevant studies was carried out independently by two researchers with didactic experience (PG, MPharm and AR, Master of Dietetics) in three step eligibility assessment process compliant with PRISMA Statement Extension for Scoping Reviews [24]. After the removal of duplicates, we screened titles and abstracts of identified literature. In the next step full texts have been screened. After selection of studies, we additionally reviewed the reference lists of the included full texts and checked the manuscripts citing the retrieved papers. Any disagreements on the inclusion of the study were resolved by discussion with third researcher with high level of competence in university-level teaching, research, and clinical experience (KP; MD, PhD).

## Results

**Fig. 1:**
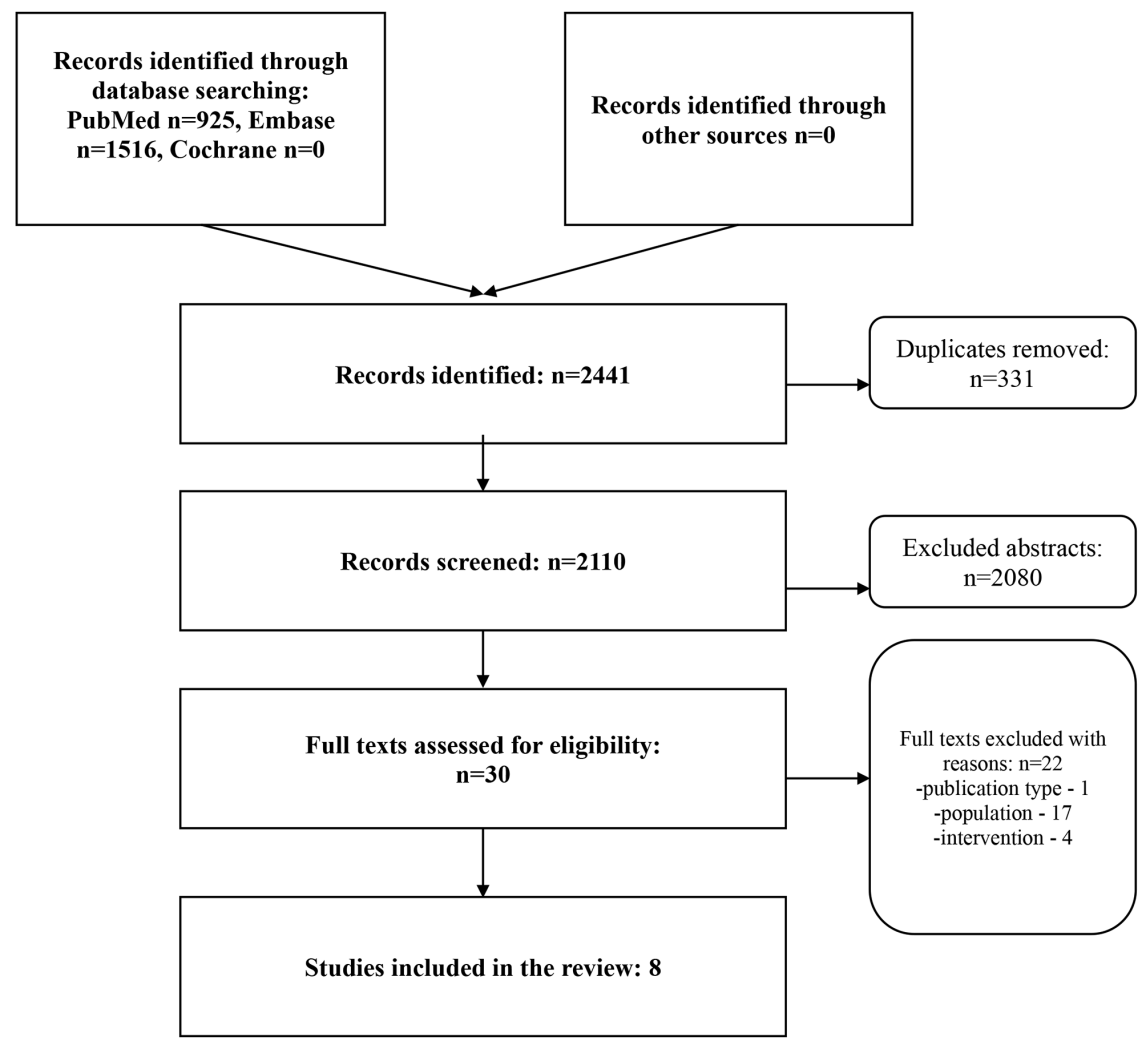
The PRISMA flowchart for the scoping review of joint interprofessional education of pharmacy and dietetics undergraduates

Of the initial 2204 records screened, we included 8 manuscripts. Details on the sources, reasons for exclusion, and selection process are presented in PRISMA diagram (Fig. 1).

In total, 234 students of dietetics and 721 students of pharmacy participated in the included studies. The characteristics of individual studies are compared in Table 1.

**Table 1:** The characteristics of the studies included in the review

Author, year	Country	Study design	Total number of participants (pharmacy students/nutrition students)	Disciplines	Setting	Teaching strategy	Topics of interest
Kent, Drysdale, Martin, Keating, 2014 [25]	Australia	Qualitative study	70 (1/13)	Dietetics, medicine, nursing, occupational therapy, pharmacy, physiotherapy, podiatry, psychology, social work, speech pathology	Voluntary University Course– Student led clinic	Service-learning (student-led clinic)	Needs of older adults recently discharged from the hospital, health screening
Wilby et al., 2014 [26]	Qatar	Descriptive survey study	42 (25/17)	Pharmacy, nutrition	University course	Case-based learning	Crohn’s Disease
Pelham et al., 2016 [27]	New Zealand	Descriptive survey study	NA (16 providers)	Dentistry, dietetics, medicine, nursing, occupational therapy, pharmacy, and physiotherapy	Clinical rotations	Clinical rotations	NA
Reitsma et al., 2019 [28]	South Africa	Qualitative study	49 (10/7)	Human movement sciences, nursing, pharmacy, dietetics, psychology, social work	Voluntary University course	Case-based learning	NA
Khalafalla et al., 2020 [29]	USA	Pre-post quantitative survey and qualitative study	15 (12/3)	Dietetics, pharmacy	Voluntary university course	Team-based learning	Obesity, healthy nutrition and lifestyle, coaching and motivational interviewing skills, cultural competency
Bhattacharya 2021 [30]	USA	Pre-post quantitative survey and qualitative study	1592 (280/5)	Dietetics, family medicine resident, nursing, occupational therapy, pharmacy, psychology, physical therapy, social welfare	24-month program, repeated twice	Team-based learning	Specific geriatrics competencies for each health profession in concordance with the “American Geriatrics Society IM-FM Residency Competencies”
van Diggelle et al., 2021 [31]	Australia	Descriptive quantitative survey study	1674 (64/33)	Dentistry, oral health, nursing, pharmacy, medicine, occupational therapy, speech pathology, physiotherapy, dietetics, diagnostic radiography, exercise physiology	Voluntary University course	Case-based learning	NA
Watts et al., 2022 [32]	USA	Pre-post quantitative survey study	856 (329/156)	Senior-level nursing students, third-year pharmacy students, senior-level nutrition/dietetics students, senior-level undergraduate, graduate social work	University course (mandatory)– student led clinic	Service-learning (student-led clinic)	NA

NA– Not applicable

### Students (majors) involved in the interdisciplinary training

Only in the studies by Wilby et al. and Khalafalla et al. did pharmacy and dietetics students have the opportunity to work together without the participation of students from other disciplines [26, 29]. Wilby et al. described a one-day course-based voluntary IPE session in which students were given a case of a patient with Crohn’s disease and aimed to develop a care plan taking into account nutritional and pharmacological issues. Attitudes towards team-based care were assessed using an adapted survey (Heinemann, Schmitt, Farrell and Brallier; 1999). The survey consisted of 11 items measuring attitudes towards interprofessional care [26, 33]. 95.1% of students agreed that the team approach improves the quality of patient care and 87.8% agreed that team meetings promote communication between team members from different disciplines. In general, the vast majority of participants agreed that interprofessional care was an applicable and beneficial concept, but there were few items in the questionnaire where opinions were divided. Controversies tended to relate to the leading role of doctors in interprofessional care and whether they had the right to interfere with patient care plans developed by other members of the healthcare team. 56.1% of respondents disagreed with the statement ‘Physicians are natural team leaders’. The other controversial items in the survey were ‘When developing interdisciplinary patient care plans, much time is wasted translating jargon from other disciplines’ (only 56.1% disagreed) and ‘Patients are less satisfied with their care when it is provided by a team’ (only 61.0% disagreed) [26]. In a study by Khalafalla et al., pharmacy and dietetics students participated in a voluntary university course aimed at improving communication between future health professionals, clarifying roles and developing teamwork skills. The authors did not specify why they chose to include these two professions in the interprofessional course. The teaching method used in this course was team-based learning (TBL) [29]. Although only pharmacy and dietetics students attended the course, the curriculum was facilitated by a team consisting of a registered dietitian, a clinical pharmacist, a paediatrician and a cardiovascular researcher. The course consisted of four sessions. Three were dedicated to theoretical knowledge on healthy eating, lifestyle and obesity, and the development of soft skills such as motivational interviewing, coaching and cultural competence. In the fourth session, students conducted mock interviews and had the opportunity to receive feedback from the registered dietitian. Student outcomes were assessed using the Interprofessional Collaborative Competencies Attainment Survey (ICCAS). In general, students’ self-perceived competencies increased in all areas assessed. In presenting the results of this study, the authors did not make a comparison between nutrition and pharmacy students [29].

In other studies, the number of majors varied from five (Watts et al.) [32] to eleven (Van Digelle et al.) [31]. The most common major to participate in an interprofessional learning environment was nursing, which was included in every study except the two that included only students of pharmacy and dietetics. Other common majors included were physiotherapy (in three studies) [25, 27, 31], social work and occupational therapy (each in four studies) [25, 27, 28, 30–32] and psychology (in three studies) [25, 28, 30]. Based on the data we obtained from the included manuscripts, none of the authors provided a rationale for the selection of specific majors.

### Learning setting and subject

The majority of the described interprofessional initiatives were implemented as university courses (voluntary or compulsory). In two studies an interprofessional clinic, where students could perform their professional roles was set (Kent et al., Watts et al.) [25, 32].One study was based in a clinical setting, as the described intervention was interprofessional clinical rotations as part of the curriculum of the participating programmes (Pelham et al.) [27].

Four studies (Kent et al., Khalafalla et al., Bhattacharya et al., Van Diggele et al.) mentioned specific learning outcomes achieved by students upon completion of the course [25, 29–31]. Six of the included studies defined subject areas (e.g., childhood obesity) or skills that students were expected to develop through participation in a course (e.g., cultural competency). In the study by Kent et al. students worked in an outpatient clinic for older adults, and the study aimed to report learning outcomes related to interprofessional collaboration in this specific setting [25]. In the study by Wilby et al., nutrition and pharmacy students worked on a case study of a patient with Crohn’s disease, but no learning objectives or specific topics were mentioned. In the study by Reitsma et al. no specific learning outcomes were mentioned, but the authors mentioned that the case studies used during the course reflected patients referred to their local clinics, e.g. patients with cancer, human immunodeficiency virus, Alzheimer’s disease, a teenager with an eating disorder and older adults. The project was planned with the involvement of a multidisciplinary team of teachers from six different health professions [26]. In the study by Khalafalla et al. the learning outcomes were defined but only related to the different aspects of nutrition education and motivational interviewing and not to the interdisciplinary practice of pharmacists and dietitians. Topics covered in the course included obesity, healthy nutrition, and lifestyle, coaching and motivational interviewing skills, and cultural competency [29]. The study by Bhattacharya et al. was part of the Geriatrics Champions Programme (GCP), a multidisciplinary project designed to train health professionals in different aspects of geriatric care. Thirty learning objectives were divided into eight domains: special considerations in geriatric care; medication management; cognitive, affective and behavioural health; complex or chronic illness in older adults; palliative and end-of-life care; hospital patient safety; transitions of care; ambulatory care. The domains were based on the American Geriatrics Society Internal Medicine-Family Medicine (IM-FM) Residency Competencies. Learning objectives within each domain were adapted for each specialty involved in interprofessional learning [30]. In study by van Diggele et al. three learning outcomes related to interprofessional collaboration were defined. The manuscript lacked in information on specific topics covered during the course [31]. Studies by Watts et al. and Pelham et al. lacked in information on learning outcomes provided by described courses [27, 32].

### Learning approach

Two of the included studies (Kent et al., Watts et al.) used the service-learning (SL) method [25, 32]. Service-learning is a learning approach that combines theoretical knowledge gained in an academic setting with practical outcomes that benefit community members in some way. The important parts of service-learning are established learning objectives that meet the needs of the beneficiaries, reflection on the learning experience, reciprocity between beneficiaries and learners so that both parties have the opportunity to learn and teach, and structuring of the learning experience [34]. In Kent et al. and Watts et al. studies SL was used to create student-led clinics. In a study by Kent et al., the student-led clinic aimed to address the needs of senior citizens being discharged from hospital. Students from different disciplines formed interdisciplinary teams and provided advice and, if an unmet health need was identified, wrote a recommendation to the patient’s GP. After each day students presented each case study to other participants [25, 32]. The study by Watts et al. aimed to compare face-to-face mobile community clinics run by students from different professions with the experience of a virtual student-run clinic. While the online clinic sessions were conducted using case studies and real patients were not present during the course, the face-to-face mobile clinics involved community members, particularly underserved older adults, and were offered in assisted living and senior centres. During the patient’s visit to the clinic, students collected health and dietary information, carried out supervised medication reconciliation and assessed the need for social support services. Debriefing sessions were held after the clinics to allow students to discuss the impact of interdisciplinary medical practice [32].

Case-based learning was the main intervention described in three of the included manuscripts (Wilby et al., Reitsma et al., van Diggele et al.) [26, 28, 31]. Case-based learning is a structured teaching approach that aims to prepare students for the future practice using clinical cases [35]. In the study by Reitsma et al., students participated in weekly meetings to discuss treatment approaches from the perspective of different health professions. The authors aimed to assess team dynamics and identify students who took on leadership roles during the intervention, as the course lasted 4–6 weeks. The number of nutrition and pharmacy students who took a leadership role during the meeting increased between the first and last meeting of the course [28]. In the van Diggele et al. study, students were asked to solve a case study and produce a video of their case management and treatment plan for this particular patient. The results of an intervention were evaluated using thematic analysis of the qualitative data. The following themes were identified in students’ responses to an open-ended question “What was most beneficial to your learning?“: opportunity to practice working in an interprofessional team, peer learning and collaboration (for both dietetics and pharmacy students), role clarification (for pharmacy students), perspectives of other disciplines in patient management (for dietetics students) [31].

The study by Khalafalla et al. used the team-based learning method, previously defined in this article [29]. The main components of this teaching approach are individual student preparation, individual and team Readiness Assessment Tests (tRATs), and in-class assignments requiring team-based decision making [36]. The second manuscript that described an intervention based on a team-based learning approach was the study by Bhattacharya et al. The intervention studied was a 24-month course in geriatrics led by facilitators from different faculties. The sessions were structured and consisted of individual and team readiness assessment tests, case studies, discussions and feedback. Before each session, students had access to online materials such as articles and patient cases. Participation in discussions and other activities was rewarded with points, and the team with the highest score received a prize at the end of the academic year [30].

### Measure of outcomes

Two studies used qualitative methods to assess the outcomes of the educational intervention delivered. (Kent et al., Pelham et al.) [25, 27]. The majority of included studies used both qualitative and quantitative approaches to the effectiveness and/or usefulness of the intervention for learners. (Reitsma et al., Khalafalla et al., Bhattacharya et al., Van Diggele et al.) [28–31]. In the Watts et al. and Wilby et al. studies, the only tool used to measure outcomes was a validated questionnaire [26, 32]. One study analysed clinical workplace providers’ experiences with IPE (Pelham et al.) [27], one (Kent et al.) mentioned both students’ and educators’ perspective, while other focused on students’ experiences [25]. A comparison of the included studies in terms of used measures of outcomes is presented in Table 2.

**Table 2:** Measures of outcomes used in the studies included in the review

Study	What was assessed	Tool	Validated tool (yes/no)
Kent et al., 2014 [25]	Comprehensive patient-centered care, role clarification, teamwork, verbal and written communication skills	Thematic analysis	No
Wilby et al., 2014 [26]	Attitudes towards team-based care	Questionnaire by Heinemann, G.D., Schmitt, M.H., Farrell, M.P., & Brallier, S.A.	Yes
Pelham et al., 2016 [27]	Reasons for participation, difference in understanding interprofessional education before and after the project, satisfaction with organizational aspects, students’ engagement, advantages and disadvantages of the project for community members	Thematic analysis of workplace providers’ perceptions on their participation in The Tairāwhiti interprofessional education	No
Reitsma et al., 2019 [28]	Overall students’ experience, perception of leadership, development of competencies	Analysis of students’ reflective journals and additional Likert-type Questionnaire	No
Khalafalla et al., 2020 [29]	Student self-perceived development of the following competencies: communication, collaboration, roles and responsibilities, collaborative family − or patient-centered approach, conflict management, team functioning	Interprofessional Collaborative Competencies Attainment Survey (ICCAS) Post-training reflection worksheet	Yes (ICCAS)
Bhattacharya et al., 2021 [30]	Overall students’ experience	Evaluation after each session (Likert scale and open-ended questions) Post-program evaluation	No
Van Diggele et al., 2021 [31]	Overall students’ experience, outcomes of the program	Questionnaire Thematic analysis of students’ written experiences	No
Watts et al., 2022 [32]	Student self-perceived development of the following competencies: communication, collaboration, roles and responsibilities, collaborative family − or patient-centered approach, conflict management, team functioning	Interprofessional Collaborative Competencies Attainment Survey (ICCAS)	Yes (ICCAS)

ICCAS - Interprofessional Collaborative Competencies Attainment Survey

## Discussion

We identified eight manuscripts relating to the described interprofessional learning for dietetics and pharmacy students. Of the included studies, two focused exclusively on dietetics and pharmacy students. Clinical teaching (particularly including geriatrics, gastroenterology, obesity, infectious diseases, oncology), cultural competence and interprofessional collaboration were identified as areas where interprofessional learning for dietetics and pharmacy students could be considered useful. However, some of the included studies did not identify specific learning objectives that would be useful in optimising future collaborations between pharmacy and nutrition or dietetics students. The included studies varied in setting, methodology and outcome measures and covered a wide range of topics relevant to clinical practice. In the included studies, case-based learning was the most commonly used teaching method. The use of this approach allows students from different disciplines to be involved in the care of the patient within their area of expertise, while encouraging interdisciplinary discussion of case management.

In the study by Wilby et al. [26], which included only dietetics and pharmacy students, authors draw attention to the important issue of involving all potential members of the interprofessional care team in interprofessional learning activities. On the one hand, such an approach would create an environment for more complex collaboration, and on the other hand, joint work between two health professions allows students to become better acquainted with the specifics of a particular health profession. What is more, in this study, only 44% of the students surveyed felt that doctors were natural leaders of the care team. It is also possible that working in teams made up exclusively of two professions allowed them to take on a significant amount of responsibility that would otherwise have been shared between team members.

This finding is in line with what was found in another study on IPE. Mei-Chi Ho et al. [37] conducted a study involving nursing and physiotherapy students. At the end of the study, the participants described a better recognition of the roles of the different health professions and how they complement each other. The students emphasised that doctors may not have sufficient knowledge of subjects that are directly related to other professions, and therefore achieved better role clarification. Similar observations about collaboration between pairs of different medical professions suggest that it may be worth exploring the potential benefits of collaboration in interprofessional, yet less diverse groups, with the aim of achieving better role awareness and encouraging communication between groups of professionals who traditionally do not share the decision-making process in patient care.

None of the papers justified why particular groups of students were included in the study. To our knowledge, there are no guidelines on this aspect of setting up interprofessional learning groups. An important observation from our review is the suggestion that when setting up classes for students of different professions, it is important to ensure that the learners are provided with educational material that allows to demonstrate the skills of each of the professions included in the study. It is also important to identify thematic areas that can be used as a basis for interdisciplinary activities. The included studies show that a variety of topics can be explored by dietetics-nutrition and pharmacy students in collaborative educational environment. The themes identified in our review where dietetics and pharmacy students collaborated were geriatrics, gastroenterology, infectious diseases and oncology, and obesity. Students also achieved learning outcomes related to cultural competence, motivational interviewing and health coaching. In the study involving only dietetics and pharmacy students, topics included managing the treatment process of a patient with Crohn’s disease and developing soft skills useful in counselling. The case of a patient with Crohn’s disease may be used to illustrate the areas in which students from these disciplines can work together. Crohn’s disease is often associated with the need for enteral or parenteral nutrition. It is essential that at least four professionals are involved in the process of managing the patient’s nutritional needs: a medical doctor, a nurse, a dietitian and a pharmacist [38]. For this reason, the management of inflammatory bowel diseases seems to be a good field for joint competence development for dietitians and pharmacists. Another area of clinical practice where interprofessional training of dietitians and pharmacists seems relevant is geriatric care, including the management of nutrition-related adverse effects of medications. It is known that anorexia [39] of ageing can be caused by some groups of prescribed and over-the-counter medications as well as polypharmacy, which causes drug-drug interactions. By working together, dietitians and pharmacists can identify the problem of loss of appetite and resolve it by suggesting deprescribing or changing the schedule of medications and meals. An education that includes the above fields allows for the systematic development of skills from the higher levels of Bloom’s Taxonomy [40] as students are not only aware of the presence of other health professions (remember), but also have the opportunity to familiarise themselves with their competencies and identify challenges that require collaboration (understand), implement protocols of practice (apply), draw conclusions on the relevance of cooperation (analyze), discuss the advantages and disadvantages of implemented solutions (evaluate), and propose innovative solutions to patient care based on the skills and knowledge of all team members (create). One of the examples of the collaboration between postgraduate dietitians and pharmacists regarding remember and understand levels of Bloom’s taxonomy is the study by Kizaki et al. [41]., in which pharmacists and dietitians were asked to rate their feelings about the availability of dietary advice in pharmacies in Japan. When surveyed, 70% of pharmacists found this type of service useful. Pharmacists also agreed that the availability of dietary advice reduces the number of medicines a patient has to take. More than 80% of pharmacists thought that the number of pharmacies offering dietary advice would increase in the future. The successful implementation of such services in Japan, followed by a satisfactory level of mutual recognition of the competences of each profession, leads to the conclusion that there is an area for collaboration between practitioners of these two professions in relation to the higher levels of Bloom’s Taxonomy model. This is also in line with the implementation of the patient-centred model of care, as integrated education at undergraduate level seems a reasonable way to build skills and awareness that are crucial for future successful collaboration between health professions to achieve high standards of patient-centred care. In such patient-centered care model, patients’ preferences, goals and beliefs take precedence over medical paternalism. This often requires a shift from a disease-centred approach, which promotes the central role of the physician, to a perspective in which other needs of the patient are considered equally important, allowing other health-related professions to take the lead. As patient treatment is often influenced by nutritional status and polypharmacy, the added value of IPE between dietitians and pharmacists would be to teach such approaches from the outset, rather than putting health professionals from different disciplines in a situation where they have to start working together as a team without proper training on how to do so. Another important component of IPE approach is promoting an inclusive attitude where uncertainties are resolved with respect for each profession and attempts are made to establish common communication practices. In such an approach, IPE is not only a teaching format aimed at the acquisition of knowledge related to the future profession, but also an opportunity for students of different disciplines to learn communication beyond the boundaries of the profession. In this way, IPE is more about giving students a space to share their thoughts, discuss and collaborate, rather than teaching them the principles of effective communication in the artificial conditions of a classroom.

The results presented by the authors of the included studies tended to focus on the overall student experience. Most projects did not use standardised assessment tools. In addition, only one study considered teachers’ perceptions of the interprofessional education experience (Kent et al.) [25] and one study considered employers’ perceptions (Pelham et al.) [27]. An important direction for further research in the area of interprofessional education of dietetics and pharmacy students seems to be not only the student experience, but also the evaluation of the educational process by experienced educators and, in later stages, by potential employers. Involving employers in the evaluation of the usefulness of interprofessional educational activities may help to identify further areas where this collaboration could have long-term benefits. Another area where further research could be undertaken is the element of evaluating the uptake of leadership by students on different courses, introduced in one of the included articles. The effectiveness of the educational interventions described could then be assessed through a shift in the perception of the relevance of one’s role in the patient care process and the willingness to take initiative and responsibility for the outcomes achieved.

The included studies represented a wide range of educational and research approaches. In view of the conclusions drawn by the authors, we have decided to summarise the implications for the further planning of joint educational and research activities for students of nutrition and dietetics and pharmacy (Table 3.).

**Table 3:** Suggested actions to improve interprofessional collaborative education and research for dietetics and pharmacy students

Suggested actions to improve interprofessional collaborative education and research for dietetics and pharmacy students	
Educational implications	Research implications
• When planning an opportunity for students from different programmes to work together, compare the curricula of courses of interest in terms of common areas and spaces where participants in activities could learn from each other, • Consider including employers’ opinions in the course development to ensure better transfer of acquired skills to the future workplace • Provide students with learning material that relates to the field of study of all the students participating, so that everyone can be involved in the learning process, • Incorporate learning methods that facilitate communication between students of different majors, • Collect feedback from all the groups included in IPE activity (including teachers, students, employers) as a source of insight for further courses improvement and implementation.	• Discuss the rationale and provide justification for the inclusion of specific major student groups in the study, • Establish (*a priori*) valid outcome measures to ensure that the outcomes are tailored to reflect the specific nature of the soft skills or professional competences that learners are expected to acquire, • Use experimental approach where possible, • Consider preceding the quantitative study with a qualitative analysis, • Collect participant feedback as a source of insight for further improvement and implementation.

Our scoping review needs to be considered in the context of its limitations. All included papers provided information on the type of learning project evaluated. However, only 4 of them (Kent et al., Khalafalla et al., Bhattacharya et al., Van Diggele et al.) reported specific learning outcomes. These outcomes differed significantly between studies [25, 29–31]. In other studies authors included only a description of the skills (Wilby et al.) [26] or competencies (Reitsma et al.) [28] that the students should acquire during the training, but these were not specified or comparable between studies.

## Conclusions

Several published studies addressed the issue of IPE jointly in pharmacy and dietetics-nutrition. The studies ranged in setting, methodology and outcome measures. Although the topics of educational courses varied, most of the included studies used case studies as the main teaching method during the courses described, two of the studies used student-led clinics and other types of problem-based learning. All of the teaching strategies used focused on students taking action and being encouraged to work together. The IPE, as delivered in the included studies, was feasible and was providing measurable benefit. The students who took part experienced improved skills both in individual soft competences and teamwork.

Changing paradigms of patient care lead to changes in educational approaches. Despite methodological differences, the reviewed papers suggest that IPE is a viable educational option. Its implementation can facilitate teamwork that is better adapted to the changing needs of the patient and thus lead to improvements in patient care. The main challenge to the wider use of IPE among students of dietetics-nutrition and pharmacy appears to be the lack of scientific evidence to support the decisions needed to carefully plan and implement IPE activities. However, the available data suggest that IPE in these programmes is feasible in a variety of settings and can be beneficial for learners.

## Data Availability

All data generated or analysed during this study are included in this published article and its supplementary information files.

## Abbreviations

WHO: World Health Organization
IPE: Interprofessional education
CAIPE: Centre for the Advancement of Interprofessional Education
TBL: Team-based learning
ICCAS: Interprofessional Collaborative Competencies Attainment Survey
GCP: Geriatrics Champions Program
IM-FM: American Geriatrics Society Internal Medicine-Family Medicine
SL: Service-learning
tRATs: Team readiness assurance tests
